# P-1714. Prevalence and Surveillance of Drug Resistance Pattern among Candida Albicans

**DOI:** 10.1093/ofid/ofaf695.1886

**Published:** 2026-01-11

**Authors:** Azizullah Khan, Mahreen Wali, Urooj Zafar

**Affiliations:** Dow University of Health Scineces, Karachi, Sindh, Pakistan; University of Karachi, Karachi, Sindh, Pakistan; University of Karachi, Karachi, Sindh, Pakistan

## Abstract

**Background:**

Candida albicans, a commensal yeast and an opportunistic pathogen, poses a global health concern due to increasing antifungal resistance and biofilm formation. This study aims to assess antifungal resistance and biofilm formation in clinical C. albicans from invasive and non-invasive cases in tertiary care hospitals in Karachi, Pakistan.

**Methods:**

Clinical C. albicans samples were collected from Civil Hospital and Aga Khan Hospital, Karachi, with respective demographic and clinical histories. Isolates were cultured on Sabouraud’s Dextrose Agar (SDA) and confirmed via Germ Tube Test (GTT). Antifungal susceptibility testing (AFST) against fluconazole, voriconazole, and caspofungin was conducted using the disk diffusion method. Biofilm formation was assessed using a qualitative ring tube method. Minimum inhibitory concentrations (MICs) for different antifungals and the clonality of C. albicans isolates will be examined.

**Results:**

A total of 202 C. albicans samples were collected and observed, all were GTT positive. These samples were subjected to AFST as per CLSI guidelines and the analysis revealed that 10% of the isolates were resistant to fluconazole, while 11% exhibited resistance to voriconazole. In contrast, all isolates remained fully susceptible to caspofungin. Underlying conditions included diabetes, hypertension, obesity, and asthma. Biofilm formation testing demonstrated that 56% of tested isolates were strong biofilm producers, while 32% formed moderate biofilms.

**Conclusion:**

The study highlights the emerging azoles resistance and biofilm formation in C.albicans.

**Disclosures:**

All Authors: No reported disclosures

